# High precision anatomy for MEG^☆^

**DOI:** 10.1016/j.neuroimage.2013.07.065

**Published:** 2014-02-01

**Authors:** Luzia Troebinger, José David López, Antoine Lutti, David Bradbury, Sven Bestmann, Gareth Barnes

**Affiliations:** a Wellcome Trust Centre for Neuroimaging, University College London, 12 Queen Square, London, WC1N 3BG, UK; b Sobell Department for Motor Neuroscience and Movement Disorders, University College London, UCL Institute of Neurology, Queen Square, London, WC1N 3BG, UK; c Electronic Engineering Department, Universidad de Antioquia, Medellín, Colombia; d Laboratoire de recherche en neuroimagerie, Département des neurosciences cliniques, CHUV, University of Lausanne, Lausanne, Switzerland

**Keywords:** Magnetoencephalography, Spatial resolution, Coregistration, Longitudinal MEG, Head movement, MEG, MSP, 3D printer

## Abstract

Precise MEG estimates of neuronal current flow are undermined by uncertain knowledge of the head location with respect to the MEG sensors. This is either due to head movements within the scanning session or systematic errors in co-registration to anatomy. Here we show how such errors can be minimized using subject-specific head-casts produced using 3D printing technology. The casts fit the scalp of the subject internally and the inside of the MEG dewar externally, reducing within session and between session head movements. Systematic errors in matching to MRI coordinate system are also reduced through the use of MRI-visible fiducial markers placed on the same cast. Bootstrap estimates of absolute co-registration error were of the order of 1 mm. Estimates of relative co-registration error were < 1.5 mm between sessions. We corroborated these scalp based estimates by looking at the MEG data recorded over a 6 month period. We found that the between session sensor variability of the subject's evoked response was of the order of the within session noise, showing no appreciable noise due to between-session movement. Simulations suggest that the between-session sensor level amplitude SNR improved by a factor of 5 over conventional strategies. We show that at this level of coregistration accuracy there is strong evidence for anatomical models based on the individual rather than canonical anatomy; but that this advantage disappears for errors of greater than 5 mm. This work paves the way for source reconstruction methods which can exploit very high SNR signals and accurate anatomical models; and also significantly increases the sensitivity of longitudinal studies with MEG.

## Highlights

• We introduce MEG coregistration scheme using 3D printed subject specific head casts.
• Using this scheme reduces relative/absolute coregistration errors to 1–2 mm levels.
• The ability to reposition between sessions results in high SNR data sets.
• At this level of coregistration error, we see a clear benefit for using individual cortical meshes.

## Introduction

Magnetoencephalography (MEG) offers many advantages over other scanning techniques. One of these advantages is the relatively high speed of measurement and subject comfort. The negative side of this pleasant scanning experience is that a large amount of co-registration error (uncertainty in head position) is introduced. This affects the forward modelling stage — the step used to relate current flow on the cortical surface to the signal measured by the sensors. Although this error is typically of the order of 5–10 mm it has a significant and detrimental effect on the estimate of electrical activity (Hillebrand and Barnes, 2011, Lopez et al., 2012). For example, investigating the effect of source extent estimation in MEG using beamformers, Hillebrand and Barnes (2011) concluded that when the cortical surface is known accurately, cortical surface models give accurate predictions of spatial extent of activation. However, if co-registration error exceeds 2 mm, this is not the case.

Typically, co-registration is performed using just 3 fiducial coils, which are placed on anatomical landmarks by the researcher (Gross et al., 2013). This introduces error, because the fiducial coil placement depends on the individual researcher's interpretation of these landmarks in each subject (both in the anatomical scan and in the flesh). One of the main drawbacks of fiducial-based co-registration is the small number of points available for matching. However, since the head does not offer a lot of sufficiently distinct landmarks, it is difficult to increase this number. There have been several efforts in the past to improve co-registration. These include bite bars (Adjamian et al., 2004) with a small number of unequivocally defined points in both coordinate frames, or surface matching techniques, to match the many points on the subject's scalp surface (as measured by a digitisation device) to that of the structural MRI (Whalen et al., 2008). Typically however both approaches give rise to similar errors of the order of 4–5 mm for different reasons— the bite-bar because all of the fiducial points are at the front of the head (and small errors are magnified); the surface matching because the head is round and smooth giving the optimisation a rather shallow cost function and it in turn relies on keeping the subject's head still for the digitisation process. Furthermore, the accuracy of the surface point measuring device used further limits the resolution of such techniques (for example although systems like Polhemus are very accurate this can be undermined by small movements in the reference sensor which often needs to be fixed to the head).

The other source of error, which affects the data quality as well as the modelling is within session head movement (again of the order of 5–10 mm). This can be corrected for in software (de Munck et al., 2001, Uutela et al., 2001, Wilson, 2004, Taulu and Simola, 2006), but entails certain assumptions and an inevitable dimension reduction.

If one could improve between session repositioning of the subject then it would also be possible to build up very high signal to noise ratio sensor level data by scanning the same individual on a number of occasions. Recently, Stolk et al. (2013) introduced a method using continuous head localization during scanning, with an option of incorporating this information in the offline data analysis. They exploited this head localization method to reposition subjects at onset of scanning between sessions, reducing intersession head distances up to 2 mm. Interestingly, they found a significant source of error in the form of a slow but steady drift away from the initial head position during the recording sessions.

It is often head movement considerations that constrain the amount of time devoted to an MEG scanning session. Ultimately, these sources of error, compounded with the uncertainties in the inverse problem lead to the rhetoric that MEG has poor spatial resolution.

In this paper, we introduce a new technique based on subject-specific head casts made using a combination of optical scans/MRI scans and 3D printing to constrain head movement. They limit between and within session movement and provide a fiducial reference frame in the form of MRI visible markers. The paper begins with a description of the construction of the head-cast and the procedures required to co-register the data. We go on to make empirical estimates of reproducibility of head position. We then make an estimate of brain movement based on repeatability of evoked response measures over several sessions. Finally, we show that the anatomical information is accurate to such a degree that we are able to distinguish between cortical surfaces using the MEG data alone.

## Methods and materials

### Head cast

**Fig. 1:**
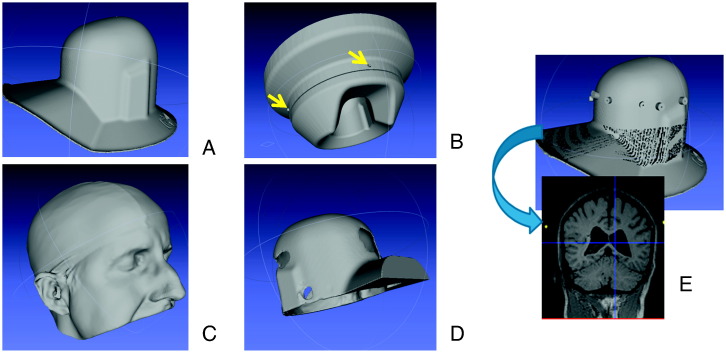
*Head cast/coregistration.* Panel A shows the optical scan of the dewar helmet, which, in combination with an optical scan of the subject's head (Panel C), serves as the basis for creating the template of the head cast (Panel D). For coregistration purposes, MRI visible markers are placed on the outside of the dewar helmet, such that their position in an MRI structural image can be related to its counterpart in an optical scan (Panel E). To obtain a transform which allows us to relate MEG sensor space and MRI space, we use the 4 measurement coil positions in relation to the MEG system dewar in a manufacturer supplied image (Panel B).

Fig. 1 shows the major components in the process of construction of the head cast. We used a manufacturer supplied dewar-helmet (A) with the same internal dimensions as the MEG dewar (typically used to check that a subject would fit within the MEG scanner). A manufacturer supplied surface image of our MEG dewar (B) was used in order to link the helmet (A) to the four reference points on the exterior of the MEG system (yellow arrows). The subject-specific head-cast was designed to fill the space between the subject's scalp (C) and the inside of the dewar-helmet (A). During the anatomical MRI scan, the subject wore both the head-cast and the dewar-helmet. The outer surface of the dewar helmet contained 12 MR visible markers that were also optically digitized (E).

### MRI scanning

MRI data was acquired using a Siemens Tim Trio 3 T system (Erlangen, Germany). The subject lay in the supine position wearing the head-cast fit into the dewar-helmet. Two types of cylindrical markers with different diameters (4 mm/8 mm; depth 10 mm, with wall thickness 1 mm of these measurements for side, top and bottom) were made using the 3D printer and filled with a copper sulphate water solution (concentration: 1 g per litre). The solution was mixed with agar to give it a gel-like consistency, minimizing leaks and making the filling of the cylinders easier. The cylinders were glued to the outside of the dewar-helmet in an approximately circular arrangement (Fig. 1E). The body-transmit coil was located inside the bore of the scanner for detection of the MRI signal. The MRI data was acquired using a 3D FLASH sequence for optimal scanning efficiency (Frahm et al., 1986). The following acquisition parameters were used: field-of view: (256,256,208) mm along the (phase (A–P), read (H–F), partition (R–L)) directions, image resolution: 1 mm^3^. The repetition time TR was set to 23.7 ms and the excitation flip angle was set to 20° to yield good T1-weighted contrast, standard in most anatomical applications (Helms et al., 2008). 8 echoes were acquired following each excitation and averaged offline to produce one anatomical image with optimal signal-to-noise. Potential sources of error from the MR acquisition should be carefully considered. A high readout bandwidth was used to preserve brain morphology and no significant geometric distortions are expected in the images. Padding was used to minimize subject motion but some residual effects might remain present in the MRI images. These effects might be further reduced by use of navigator echo techniques. The total acquisition time was 21 min 07 s.

### Optical scanning

We obtained an optical scan of the subject's head/face anatomy (Fig. 1C), using an Artec 3D handheld optical scanner (http://www.artec3d.com/3d_scanners/artec-mht). This type of scanner has a resolution of up to 0.5 mm, and captures images at a rate of 15 frames per second. It has a 3D point accuracy of up to 0.1 mm. The handheld scanner is moved around the subject's scalp at a constant, moderate speed, whilst the subject's head is kept still using a bite-bar (Adjamian et al., 2004). It should be noted that the scanning device we used generally fails when attempting to capture images of dark, shiny objects. Also, some areas of the head and face are difficult to capture, such as ears and hair. For these reasons the subjects wore swimming caps to cover their hair. Since the ears were not particularly important for our proposed design (and also difficult to scan), we only ensured that enough space was left to accommodate them in the finished head cast. Note that swimming caps were only used at this stage, purely to avoid problems with the optical scanner, these caps had a thickness of 0.27 mm and therefore served mainly to constrain the hair without adding appreciable thickness to the scalp estimate.

In order to fit this image to the MEG system dewar surface, a similar scan was obtained of the inside of the MEG dewar-helmet. Following this, we also scanned the outside of the dewar-helmet with MR visible markers attached (panel E). We used the Magics (http://software.materialise.com/magics-0) CAD package to process the images.

### Printing

The images of the internal dewar surface (Fig. 1A) and the scalp (Fig. 1C) were aligned by eye to place the subject's head in a typical recording position. These two surfaces were converted into solids and the Boolean difference between the two structures was used to make the head cast (Fig. 1D). However, a few practical issues had to be considered: to ensure as tight a fit as possible, the head cast had to have some ‘anchor’ points, consisting of distinct anatomical marks — such as the inion bone or the bridge of the nose. We therefore decided to let the cast come down past the eyes, covering the bridge of the nose. In order to allow the subject to put on and take off the helmet, the image was split vertically down the centre, incorporating a simple locking mechanism. This image was passed on to the 3D printer. For our prototype, we used an ZPrinter 350, which has a resolution of 350 × 400 dpi (dots per inch).

### Co-registration

The following process consists of 3 distinct registration stages. We wish to estimate the transformation from MRI to dewar-helmet space; dewar-helmet space to MEG dewar space and finally MEG dewar space to MEG measurement space. Given all these stages we can then produce a single rigid body transformation between MRI anatomy and MEG sensor space.

In each case we used the ICP (Iterative Closest Point) algorithm to match point clouds (http://www.mathworks.co.uk/matlabcentral/fileexchange/24301-finite-iterative-closest-point).

#### Native MRI to dewar-helmet

The anatomical MRI scan gives an image showing the position of the cortex relative to the registration markers (MRI space) (Fig. 1E). These registration markers were extracted from the MRI scan through a simple thresholding operation. A similar process was performed to extract the locations of the registration markers in the optical image. This resulted in two point clouds which were aligned using the ICP algorithm outlined above.

#### Dewar-helmet to MEG dewar

The next step is to transform from the (optical) dewar-helmet space to the MEG dewar space (the external surface of the MEG measurement system). Again, we need to find a set of appropriate reference points. Since the dewar-helmet is an exact replica of the inside of the MEG dewar, we can use the subset of points of the manufacturer supplied data (Fig.1, Panel B) that describes the same shape. We then follow a similar procedure as outlined above, bringing the images into approximate alignment, then following this up with ICP alignment (Fig. 1, Panel B).

#### MEG dewar to MEG space

In the last stage, we need to acquire a transformation matrix between MEG dewar space and MEG sensor space. The relationship between these two spaces will depend on the locations of the sensors within the measurement dewar. In order to do this we matched the four coil calibration points based on the MEG dewar specification (arrows in Fig. 1B) to those measured using the MEG sensors to locate fiducial coils attached to these reference locations (we also accounted for coil thickness in these estimates). We made 5 measurements of each coil position giving rise to four point clouds to fit to four single points.

#### Bootstrapping for accuracy

In order to make an estimate of the robustness of these various fitting procedures we bootstrapped (using 100 bootstrap iterations) each fitting stage independently and then all fitting stages at once. Each of these 100 iterations gave rise to a different transformation matrix that we used to estimate a new fiducial location (on the subject's scalp). This distribution of points gave a measure of the expected variance in fiducial location due to measurement and fitting error. In a first step, bootstrapping was applied to a single transformation stage in turn, whilst all others were held constant. Next, bootstrapping was applied to all transforms simultaneously, to get an estimate of the likely absolute coregistration error.

#### Testing for reproducibility

In order to test for within and between run reproducibility in head position and head-cast position we attached one fiducial coil to the cast and one firmly to the subject's head (just above the right ear). Over a ten minute period, we made eight recordings of the coil positions whilst the subject was seated within the dewar. Over the subsequent forty minute period we then made eight further measurements, of these coil positions but removing the subject from the dewar and the head-cast in between runs.

### Empirical validation

We have now performed the same experiment on subject 1 nine times over a six month period. We wanted to use this functional data, based on the location of the cortex, to corroborate our estimates of co-registration error based on the scalp surface.

#### Subject task

We used a simple finger movement task adapted from Muthukumaraswamy (2011), which involved simple abductions of the right hand index finger performed to an auditory cue. The cue consisted of a simple auditory tone (1000 Hz), played via a piezo electric device connected via plastic tubing to ear-inserts, followed by an inter-stimulus interval of 3.4–4.5 s. This gave approximately 130 epochs of data per ten minute recording session. EMG traces of the first dorsal interosseus (FDI) were used to track finger movements (although we did not make use of this information directly for the purpose of this paper). Each session of scanning was split into 4 sections of 10 min each. During the first ten minutes, the subject was told to perform simple abductions of the right hand index finger on hearing the pip. Following these 10 min of task performance was a rest period of equal length, during which the subject was simply told to remain as relaxed as possible. This was followed by another 10 min of task performance and 10 min of rest (the rest data were not used in this study). We repeated this same recording five times over a six month period. In the first recording run we had a triggering problem and only one of the two task runs was recorded giving a total of nine task runs.

#### Source reconstruction

We used averaged evoked responses from 0 to 300 ms (0–80 Hz) time-locked to the auditory cue, baseline corrected based on − 200 to 0 ms. The only artefact removal was the removal of trials containing large jumps (due to loss of lock in the feedback electronics) which could be clearly seen by eye (as the subject was unable to open their eyes we had no need to correct for eyeblinks). These data were projected into 100 (for the majority of this work) or 250 (for Fig. 6) orthogonal spatial (lead field) modes and 16 temporal modes. We used the greedy search option of the MSP algorithm (Friston et al., 2008) implemented as outlined in Lopez et al. (2012). The MSP algorithm requires a set of covariance matrix priors corresponding to cortical patches to be defined a-priori. We used a pseudo-random (the same sequences were used when comparing different surface models) selection of 512 mesh vertices to define the patch centres and produced 32 such solutions (each based on different patch sets) per dataset. There were no symmetric priors used. The MSP algorithm returns a Free energy value which approximates the model evidence for the final generative model. This generative model includes the cortical surface used and the lead fields and therefore model evidence can be used to select between different models of the geometry (Henson et al., 2009, Lopez et al., 2012). We reconstructed the same MEG data onto two cortical surfaces: the subject's cortical surface or the canonical mesh (Mattout et al., 2007). The canonical mesh is simply an extracted mesh from a template brain in standard space warped into the subject space. The algorithm returns posterior estimates for the mean and variance of current density at each cortical location. These posterior estimates can be converted into either posterior probability maps (probability that current density is non-zero) or plots showing the mean and 95% confidence intervals (± 1.66* posterior standard deviation).

## Results

### Absolute co-registration error

**Fig. 2:**
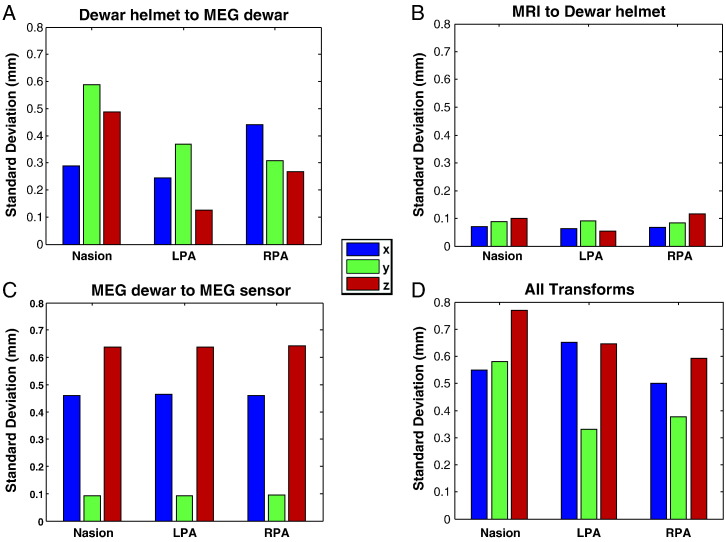
*Evaluation of repeatability of co-registration results by means of bootstrapping.* The bars show the standard deviation the three fiducial locations in x, y and z directions based on bootstrapped estimates of the transformation matrices. Panel A shows the likely error in fitting the dewar-helmet shape (Fig. 1A) to the MEG dewar (Fig. 1B). Panel B shows the fitting of the MRI fiducial locations to the dewar-helmet fiducials (Fig. 1E). Panel C shows an estimate of the error due to fitting the MEG sensor space (as measured using fiducial coils) to MEG dewar fiducial points (Fig. 1B, yellow arrows). Finally, all transforms were bootstrapped simultaneously (Panel D) to get an idea of the total expected absolute coregistration error— which we would expect to be of the order of 1 mm per fiducial.

In this first stage we want to get an estimate of the absolute co-registration error due to the fitting process. We bootstrapped the calculation of the different transformation matrices and looked at the variability in the estimates of fiducial locations to get an estimate of absolute coregistration error. This variability was estimated per transform (Figs. 2A–C) and for all transforms simultaneously (Fig. 2D). The bars indicate standard deviations for three fiducial locations in the x (blue), y (green) and z (red) direction. We found that the transformation between the MEG dewar space and the MEG sensor space (panel C) potentially represents the biggest source of error — most likely because it was based on the smallest number of unique points. However in all cases, and in particular for the case when all stages were bootstrapped simultaneously (panel D), the expected errors in any one dimension are still within the sub-millimetre range.

Note in Fig. 2C (MEG dewar to sensor) there is less error in the y than x and z directions. This transformation is the matching of 4 fiducial markers on the dewar surface with 4 fiducial coil locations (estimated by the MEG system). As we were working with a set of 3 fiducial coils we measured left, right and front for half the runs; and left, right and back for the other half. This meant that we have twice as many points for left and right fiducials than for front and back which most likely gives rise to the increase in precision in the y (left–right) direction. The average distance of an MEG estimated fiducial location to its corresponding point on the manufacturer supplied dewar surface was 0.23 mm.

### Relative co-registration error

We used two subjects to test for within and between run reproducibility in head position and head-cast position. In each case, we attached one fiducial coil to the cast (close to the left pre-auricular) and one firmly to the subject's head (just above the right ear). Over a ten minute period, we made eight recordings of the coil positions whilst the subject was seated within the dewar. Over the subsequent forty minute period we then made eight further measurements, of these coil positions but removing the subject from the dewar and the head-cast in between runs.

**Fig. 3:**
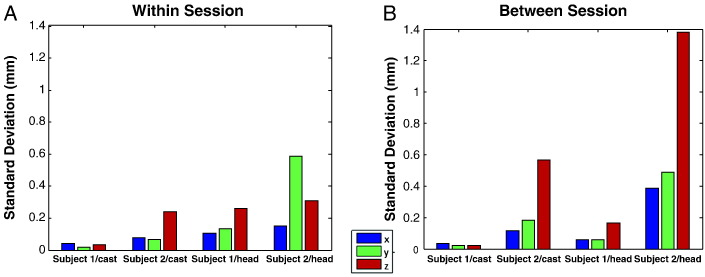
*Within (A) and between (B) session cast/head movement estimates for both subjects.* Panel A. Within session estimates of variability in cast (left most bars) and head (right most bars) position. Note both cast-movement estimates are below 0.3 mm indicating that the external surface of the casts fit securely within the MEG dewar. Also note also that for subject 1 head movement is limited to within 0.3 mm but for subject 2, whose cast fit less well internally (most likely due to the optical scanning stage, see discussion), this figure is around 0.6 mm. Panel B shows between session cast/head movement estimates for both subjects. The between session errors in cast location were comparable with the within session errors for subject 1, but for subject 2 showed and increased to around 0.6 mm in the z (vertical) direction. In terms of head position, we were able to reposition subject 1 to within 0.3 mm between sessions. Whereas subject 2 was again more variable but could still be repositioned to within 1.4 mm, the error again predominant in the z direction indicating some freedom of vertical movement within the cast.

Fig. 3 shows the standard deviation of coil position as measured within (panel A) and between sessions (panel B). The leftmost sets of bars in each figure indicate variability in head cast position with respect to the dewar for both subjects. The head-cast location varies by less than 0.3 mm within runs; showing that the cast fit rigidly within the dewar and gives us an upper bound on the error (due to reproducibility) of the MEG system estimate of the fiducial coil locations. For the coil attached to the subject's head the expected errors are larger and give an idea of the fit of the cast to the subject's head internally. The first subject shows very little within run movement the expected error was below 0.2 mm predominantly in the vertical (z) direction. For the second subject the errors were lager yet still sub-millimetre (expected (3D) Euclidean displacement of 0.7 mm from the mean).

Fig. 3B illustrates variability in head and head cast position between sessions. Again the left-most panels shows repositioning errors on the two head-casts inside the dewar; below 0.6 mm but dominant in the vertical (z) direction (i.e. there is little rotation, but some error introduced when the cast is not pushed firmly up inside the dewar). Similarly, for both subjects, the variability of head cast position with respect to the MEG dewar (3B, right-most bars) is most prominent in the z direction, reflecting the fact that the subject (and cast) has some vertical freedom of movement, but little room to rotate. Note that the variability is larger for our second subject (1.4 mm in the z direction). Although there is some difference in cast-repositioning error (compare two left-most groups of bars) it would seem that the majority of the increase in error for the second subject is due to a relatively poor fit of the cast internally. Most likely this is due to errors in the optical scanning (see Discussion). Within and between session movement estimates are very similar for subject number one — moreover, they seem to follow the head cast. In other words, subject one's head is moving little within the cast and most of the error is due to replacing the cast within the dewar. Expressing these data as multivariate Gaussian probability distributions, this means that the expected coregistration errors (as a single Euclidean distance) are 0.29 and 1.3 mm for subjects 1 and 2 respectively with corresponding 95% confidence limits of 0.6 and 2.7 mm.

### Cortical coregistration estimates

Our previous measurements were based on relative movements of the subject's scalp within and between sessions. Ultimately, however, it is the error in the location of the cortex with respect to the MEG scanner that we wish to estimate. In this section we use functional data, recorded over multiple sessions, to make estimates of the relative and absolute cortical location.

### Relative cortical location

**Fig. 4:**
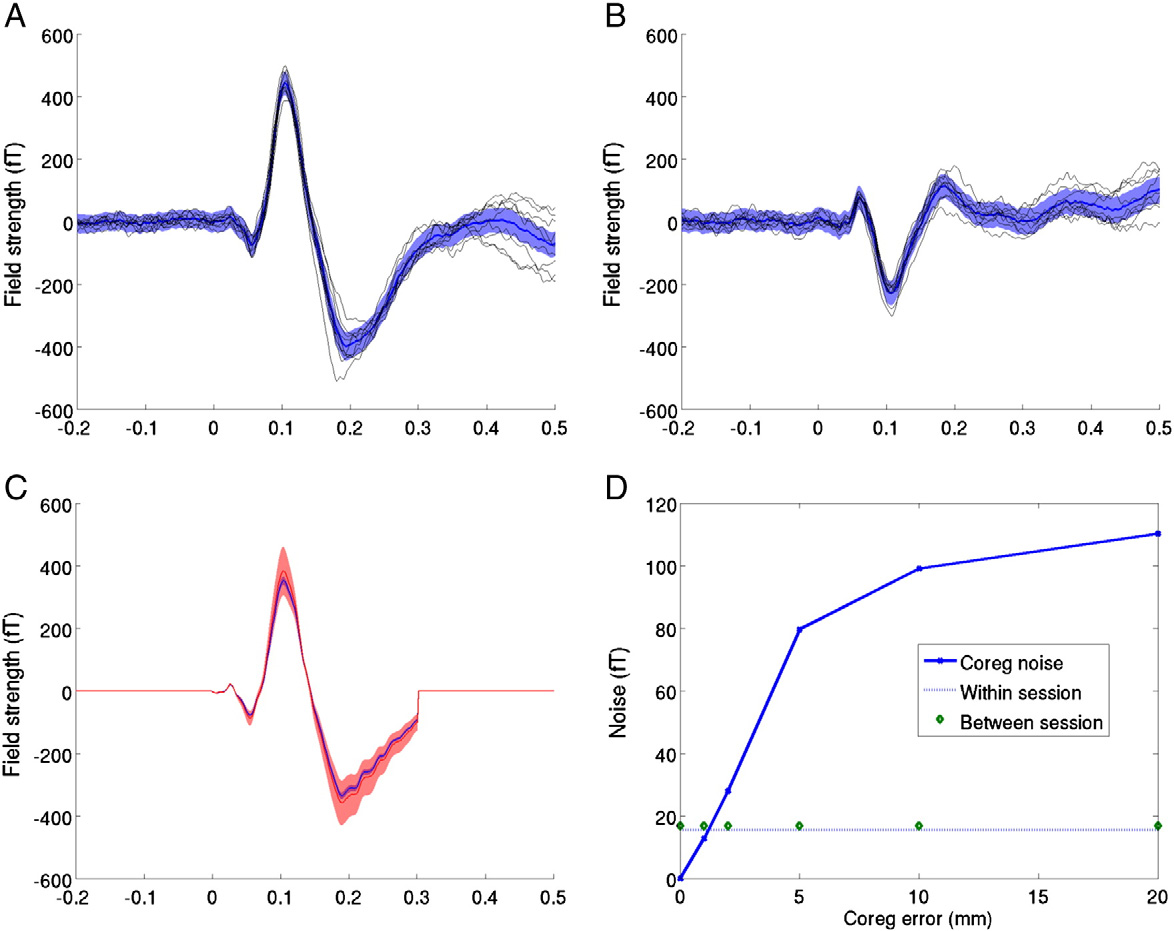
*Repeatability of between session sensor level evoked responses.* Panels A and B show the MEG channel with highest variance (MLT35) and its contralateral counterpart (MRT35) respectively. Solid black lines show the nine averaged evoked responses from each session over a six month period. Data were baseline corrected from − 0.2 to 0 s. The shaded regions show the mean within session 95% confidence intervals on this mean. Note that the between session variability in the evoked response is of the same order as the within session variability. This between session variability includes physiological changes in the evoked response as well as noise due to changes in relative head position over scans. In panel C, current density estimates from the grand average evoked response are projected back out to the sensors using different forward models. Only the time period used for the source inversion is shown (hence the zeros outside 0–0.3 s). The forward models differ in their co-registration error: the shaded blue area shows the sensor level standard deviation due to 1 mm error in coregistration over sessions; whilst coregistration errors of 5 mm (shaded red) give rise to considerably larger noise levels. Panel D shows sensor noise as a function of coregistration error. In the absence of any other noise, increasing the co-registration error from 1 to 5 mm incurs a 5 fold increase in RMS noise levels. The dotted line shows the within session standard error (the variability on the mean of any one session expected by chance) whilst the circles show the standard deviation of the evoked response (at peak latency) over sessions. Note that there is almost no difference between these two lines, which based on the simulation, should begin to diverge for coregistration errors of greater than 1 mm.

Changes in the location of the head over scanning runs will give rise to increased sensor level variability. Figs. 4A,B show the averaged evoked responses from two (the channel with maximum variance, and its contralateral partner) sensor channels over the six month scanning period. The solid black lines show the averaged evoked responses from the 9 recording sessions. The solid blue line shows the grand mean (over all 9 sessions), the shaded area shows 95% confidence intervals on this mean based on the average within (not between) session variance. Note that the variability over sessions is of the same order as the within session variance; suggesting that little additional coregistration noise has been added to the data. In order to quantify the amount of coregistration noise one would expect for different levels of coregistration error we performed a series of simulations.

### Simulation

**Fig. 5:**
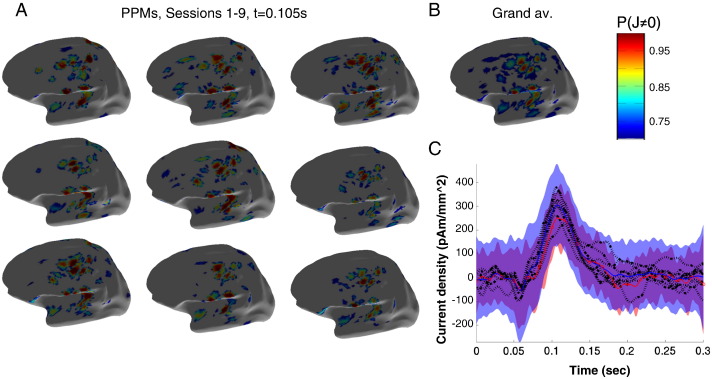
The repeatability of the MSP source level estimates. Panel A shows posterior probability maps of the current density (J) on the inflated cortical surface at 105 ms post stimulus at each of the 9 scanning sessions from within six month period. The colour scales show probability that J is non zero. Note the similarity over scanning sessions and the similarity to the MSP source level estimate based on the grand average (Panel B). Panel C shows the time-series estimates from the peak location in source space. Dotted black lines show source level estimates over the 9 scanning sessions and the average of these is shown as a blue solid line; the average 95% confidence estimates on any one session are shown in the blue shaded area. The estimate of current density on the grand mean sensor level data (red circles) and its reduced 95% confidence limits (red shaded) are also shown.

In order to estimate the amount of sensor noise one would expect due purely to coregistration error we projected a realistic current density (based on the inversion of the grand average, see next section and Fig. 5B) back through a forward model based on different fiducial locations. We took 16 random fiducial locations drawn from a Gaussian distribution of standard deviation equal to the coregistration error (0,1,2,5,10 and 20 mm) and computed the estimated sensor level signal. Fig. 4C shows the effect of co-registration errors on sensor noise levels (only the data from inverted time period 0–300 ms has been re-estimated). Solid lines show the mean and the shaded regions show plus and minus one standard deviation. For 1 mm coregistration error (blue) the standard deviation of the signal is of the order of 15fT (blue shaded); for 5 mm coregistration error (red) it is around 80fT. Fig. 4D shows the relationship between coregistration error (solid blue) and the expected sensor level standard deviation. Intuitively, the more coregistration error, the more between session signal variance. By decreasing coregistration error from 5 mm to 1 mm one can increase the sensitivity to between session effects by a factor of 5. This simulated curve (solid blue) gives us an estimate of the amount of sensor level noise one would expect due to additional between session coregistration error. For example, for between session repositioning errors of the order of 1 cm we would expect between session variability to increase by 100 fT over within session variability (assuming this is negligible). We can now work backwards to get an estimate for coregistration error based on the difference in between and within session noise levels. The two measured values corresponding to mean within session (signal) variability between session (signal) variability are shown as dots and circles respectively. Importantly, the multiple scanning sessions have hardly added to the within session noise level suggesting that minimal coregistration error has been introduced (as observed also in Figs. 4A,B). Assuming (in the worst case) that any difference in within and between session variability can be attributed solely to head movement (rather than other physiological factors), these functional estimates based on the cortex corroborate with the scalp estimates and put the between session coregistration error at the sub-millimetre level.

### Source reconstruction

Fig. 5A shows the source level posterior probability maps (PPM), for the MSP reconstructions based on a 0–300 ms window (baseline corrected based on − 200 to 0 ms), at the time of the peak evoked response over sessions on the inflated cortex. Note the consistency of these images in which no fiducial coils were used (or compare field maps at this latency shown in supplementary Fig. S1). Also apparent is the patchy nature of the reconstruction due to the discrete set of MSP source priors used. Fig. 5B shows the corresponding PPM of the MSP inversion of grand average sensor data, again consistent with all the individual runs (Supplemental Fig. S2 shows PPMs for the corresponding minimum norm reconstructions). Fig. 5C shows the current density over time estimates at the peak of this grand average PPM (at MNI coordinates: x = 34.3623 y = − 31.6975 z = 62.0449 mm). The dotted black lines show individual current estimates from each of the 9 sessions; the mean of these current estimates is shown as a solid blue line and the 95% confidence interval (expected in any one session) on this mean is shown as the blue shaded area. The MSP estimate based on the grand (sensor level) mean (red circles) closely follows this estimate, which would not be the case had any non-linearities been introduced by substantial head movement. The confidence intervals on this grand mean MSP estimate (red shaded) are decreased with respect to the signal session (blue shaded) estimates; but not by as one would expect (a factor of 3 or the square root of the number of scanning sessions). Presumably this difference is due to non-linear behaviour both at the level of the brain, and at the non-linear MSP optimisation stage.

### Absolute cortical location

**Fig. 6:**
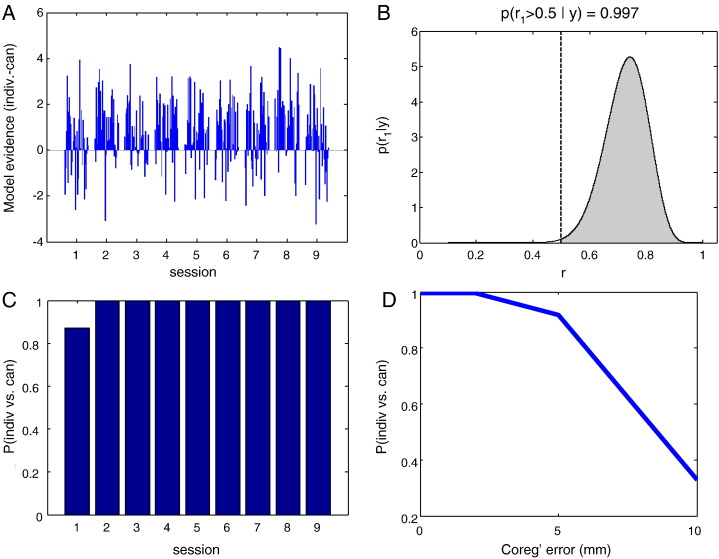
*Investigating sensitivity to cortical model used.* Panel A shows the comparison between individual and canonical surface models in terms of log model evidence for each of 32 patch(/prior) sets used to fit the averaged evoked response from 9 separate sessions. The model evidence difference is generally positive, indicating that the individual cortical surface model is favoured in the majority of cases. Using a random effects analysis, Panel B shows the frequency with which the individual white surface is favoured over the canonical model within Panel A session 9. Panel C shows the exceedance probability (the area under the curve in Panel B where frequency r > 0.5) plotted over all sessions. Again, the evidence is overwhelmingly in favour of the individual cortical surface. Lastly, in Panel D, we investigated the effect of adding additional co-registration error and performing the same analyses. As co-registration error increases, the advantage of using a model based on the individual subject's cortical anatomy diminishes to the point where it is no longer favoured over the canonical model.

Finally, in order to get some idea of whether we now have useful and precise anatomical information registered to the MEG sensors, we tried replacing the subject's cortical surface with someone else's. The use of a canonical mesh (Mattout et al., 2007) involves the use of a generic cortical mesh in standard space which is then warped into subject space. The method is attractive as it removes the need to extract per-subject cortical meshes, and has been shown to have little impact on the overall source reconstruction (Henson et al., 2009). We therefore reconstructed the MEG averaged evoked response data for each session onto both the subject's individual mesh and the canonical mesh. One of the advantages of using the Multiple Sparse Priors (MSP) framework is that it returns the negative variational free energy, an approximation to the log model evidence. Free energy will increase with improved model fit to the data, but will decrease as the model becomes more complex (i.e. the simplest models that explain most of the data are favoured). The relative likelihood of one model over another can be assessed by comparing their Free Energies. A Free Energy difference between two models of 3 or more equates to one model being 20 times more likely than the other. The model in this case includes both the forward model (cortical sheet, head location) as well as priors (MSP patches) used to explain the data (Henson et al., 2009). The difference in log model evidence between the two cortical surfaces for each of 32 reconstructions (using different patch sets) for each of 9 sessions is shown in panel A of Fig. 6. It is clear that the evidence is in favour of the individual mesh (predominantly positive). An alternative way to summarise this information is to ask if we were to draw a simulation from the set at random how often would it be in favour of the individual mesh (Stephan et al., 2009). This can be more robust than typical fixed effects analysis as it is relatively immune to outliers (a very large free energy difference in just one simulation). Panel B shows the frequency (r) at which we would chose the individual over the canonical mesh if we were to chose a patch set (from run 9 in Panel A) at random. Summing the area under the curve above 0.5 gives the probability that the individual mesh is the better model (an exceedance probability). These exceedance probabilities are then plotted per session in Fig. 6C. Note again that there is strong and consistent evidence for the individual mesh over a six month scanning period. We were interested to see how if we could reconcile this finding with previous work by Henson et al. (2009) who found no evidence for an improvement using individual meshes. We repeated the same analysis, yet this time we added different amounts of co-registration noise to our head model for each session (Fig. 6D). Note here that as the coregistration increases beyond 5 mm (typical coregistration error) there is little to distinguish between the two meshes.

## Discussion

In this paper, we have presented a new and conceptually simple approach to reduce between and within session coregistration errors in MEG. The head casts provide a robust fiducial frame reducing absolute and relative errors for coregistration to anatomy to within 1–2 millimetre levels. By constraining head movement within and between sessions they also allow us to build up very high SNR data sets. The between session repeatability improves our longitudinal sensor level sensitivity to signal amplitude change by a factor of 5. This equates to scanning 25 times fewer sessions (at 1 mm coregistration error) and retaining the same sensitivity (as at 5 mm coregistration error).

Both coil based and functional estimates put the relative coregistration error at around 1 mm. The absolute coregistration error (i.e. systematic errors between MEG cortical and true cortical location) is more difficult to assess. Here we used boostrapping to test the robustness of our estimates but this cannot account for some physiological issues: for example, the movement of the cortex within the skull between upright MEG and supine MRI scanning. With this in mind we tested whether we were able to distinguish between the subject's individual anatomy and a warped version of canonical anatomy within that same individual's skull. We were able to do this and furthermore show that adding back co-registration error destroyed this ability to discriminate. Our results explain the findings of Henson et al. (2009) who found that using a cortical model based on the individual subject's anatomical MRI does not necessary lead to better source reconstruction outcomes. However we were surprised that we had to add over 5 mm error (to the existing error) before the two models became equally likely. We can say therefore that our absolute coregistration error lies somewhere in between the bootstrap estimate (1 mm) and this upper bound.

It would seem that the biggest source of error in the head cast production process is the optical scanning— which has difficulty dealing with hair (we used a swim cap) and also difficulty in piecing together a number of smooth round surfaces. We are currently revising our procedure to use scalp surface information directly from the anatomical MRI. Also, in this first iteration no cut outs were left for the eyes, the aim being to constrain the head as much as possible. This restricted us to using only non-visual paradigms, and may also be intimidating to some subjects who are prone to claustrophobia. However, the design can easily be made to accommodate openings or periscopes for the eyes, which is a consideration for future incarnations of these head casts.

However, given the improvements using the head cast technique, it will be interesting to look into further factors which may present sources of error. For instance, error could be introduced by the fact that subjects are in the supine position for MRI acquisition, but seated upright in the MEG scanning system. The location of the cortical surface may well differ between these two positions, and more work is needed to investigate this. Furthermore, the accuracy of the anatomical MRI scan may also present a source of error. In our case, the choice of MRI sequence to be used was limited by the fact that we had to accommodate the dewar-helmet in the MRI scanner, which is too large to fit most head coils. Whilst we found the resulting image to be sufficiently accurate for our purposes, further work is needed to assess the influence and possible contribution of error of anatomical MRI resolution.

Besides the improved longitudinal sensitivity and increased precision on the underlying volume conductor model parameters, we believe a major application of this work will be to build up very high SNR datasets to help define the limits of MEG source localisation and spatial resolution. Given accurate anatomical information, and the ability to record over long periods of time, there is no theoretical limit on the potential spatial resolution achievable with MEG. In future work we will describe the use of these head-casts in order to estimate which cortical laminae give rise to specific signal characteristics.

We believe that the above characteristics will render the use of the head cast technique particularly valuable in the context of studies which depend on high quality data for a limited number of subjects, e.g. longitudinal drug studies. The method also has application in any clinical setting in which precise localisation of function or pathology is required; for example, as a tool for pre-surgical mapping in epilepsy patients. The only constraint we foresee is that the head-casts can induce feelings of claustrophobia and we are working on new less intimidating designs.

## References

[bb0005] Adjamian P., Barnes G.R., Hillebrand A., Holliday I.E., Singh K.D., Furlong P.L., Harrington E., Barclay C.W., Route P.J. (2004). Co-registration of magnetoencephalography with magnetic resonance imaging using bite-bar-based fiducials and surface-matching. Clin. Neurophysiol..

[bb0010] de Munck J.C., Verbunt J.P., Van't Ent D., Van Dijk B.W. (2001). The use of an MEG device as 3D digitizer and motion monitoring system. Phys. Med. Biol..

[bb0015] Frahm J., Haase A., Matthaei D. (1986). Rapid three-dimensional MR imaging using the FLASH technique. J. Comput. Assist. Tomogr..

[bb0020] Friston K., Harrison L., Daunizeau J., Kiebel S., Phillips C., Trujillo-Barreto N., Henson R., Flandin G., Mattout J. (2008). Multiple sparse priors for the M/EEG inverse problem. NeuroImage.

[bb0025] Gross J., Baillet S., Barnes G.R., Henson R.N., Hillebrand A., Jensen O., Jerbi K., Litvak V., Maess B., Oostenveld R., Parkkonen L., Taylor J.R., van Wassenhove V., Wibral M., Schoffelen J.M. (2013). Good practice for conducting and reporting MEG research. NeuroImage.

[bb0030] Helms G., Dathe H., Dechent P. (2008). Quantitative FLASH MRI at 3 T using a rational approximation of the Ernst equation. Magn. Reson. Med..

[bb0035] Henson R.N., Mattout J., Phillips C., Friston K.J. (2009). Selecting forward models for MEG source-reconstruction using model-evidence. NeuroImage.

[bb0040] Hillebrand A., Barnes G.R. (2011). Practical constraints on estimation of source extent with MEG beamformers. NeuroImage.

[bb0045] Lopez J.D., Penny W.D., Espinosa J.J., Barnes G.R. (2012). A general Bayesian treatment for MEG source reconstruction incorporating lead field uncertainty. NeuroImage.

[bb0050] Mattout J., Henson R.N., Friston K.J. (2007). Canonical source reconstruction for MEG. Comput. Intell. Neurosci..

[bb0055] Muthukumaraswamy S.D. (2011). Temporal dynamics of primary motor cortex gamma oscillation amplitude and piper corticomuscular coherence changes during motor control. Exp. Brain Res..

[bb0060] Stephan K.E., Penny W.D., Daunizeau J., Moran R.J., Friston K.J. (2009). Bayesian model selection for group studies. NeuroImage.

[bb0065] Stolk A., Todorovic A., Schoffelen J.-M., Oostenveld R. (2013). Online and offline tools for head movement compensation in MEG. Neuroimage.

[bb9000] Taulu S., Simola J. (2006). Spatiotemporal signal space separation method for rejecting nearby interference in MEG measurements. Phys. Med. Biol..

[bb0070] Uutela K., Taulu S., Hamalainen M. (2001). Detecting and correcting for head movements in neuromagnetic measurements. NeuroImage.

[bb0075] Whalen C., Maclin E.L., Fabiani M., Gratton G. (2008). Validation of a method for coregistering scalp recording locations with 3D structural MR images. Hum. Brain Mapp..

[bb0080] Wilson H.S. (2004). Continuous head-localization and data correction in a whole-cortex MEG sensor. Neurol. Clin. Neurophysiol..

