# Supplementary material for: High precision anatomy for MEG
Source: Neuroimage. 2014 Feb 1;86:583–91. doi: 10.1016/j.neuroimage.2013.07.065 (PMC3898940; doi:10.1016/j.neuroimage.2013.07.065)
Supplement: Supplementary file 1 — Supplementary Figures. [file mmc1.pptx]

## Slide 1
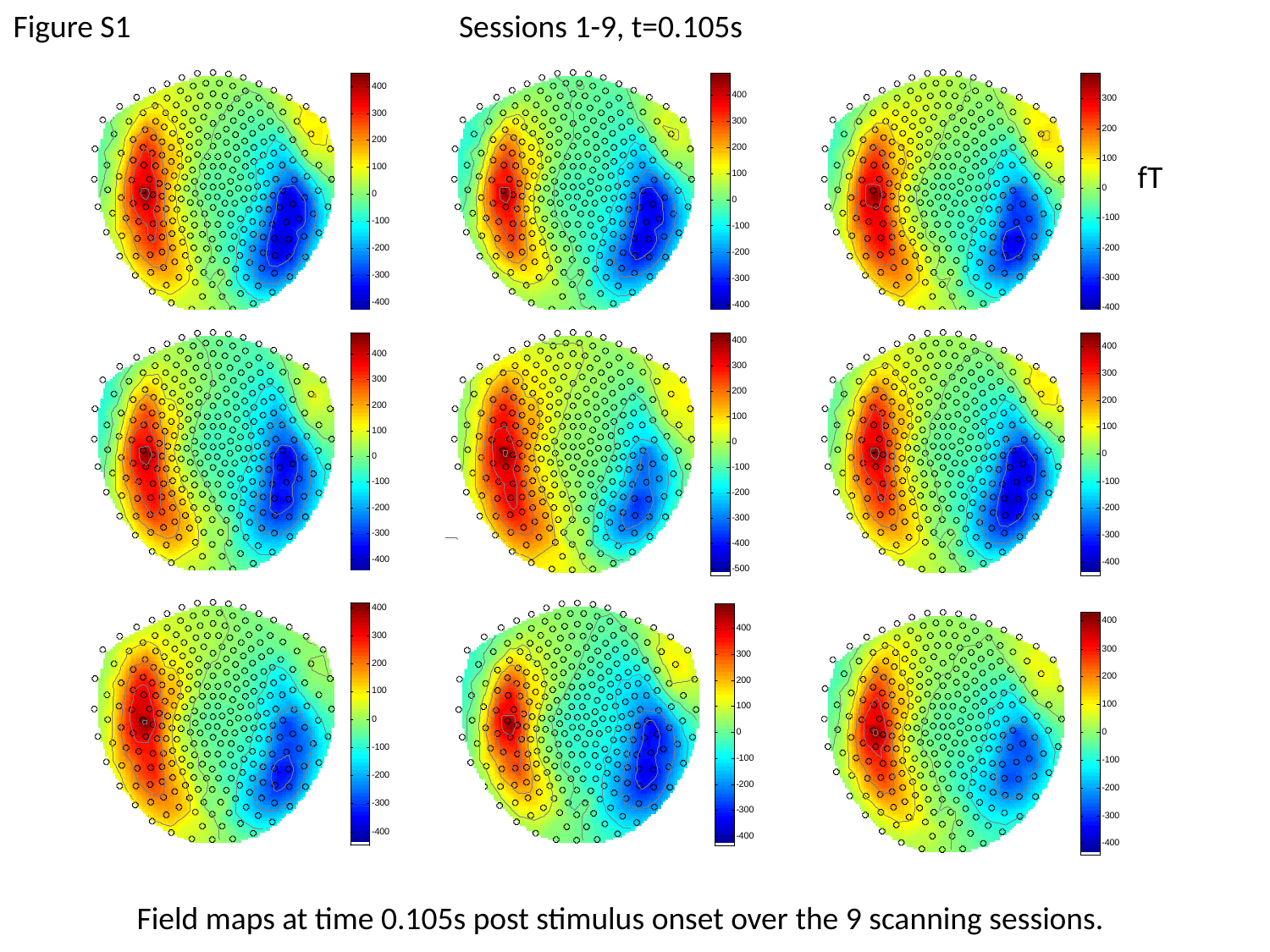

Figure S1
Sessions 1-9, t=0.105s
fT
Field maps at time 0.105s post stimulus onset over the 9 scanning sessions.

## Slide 2
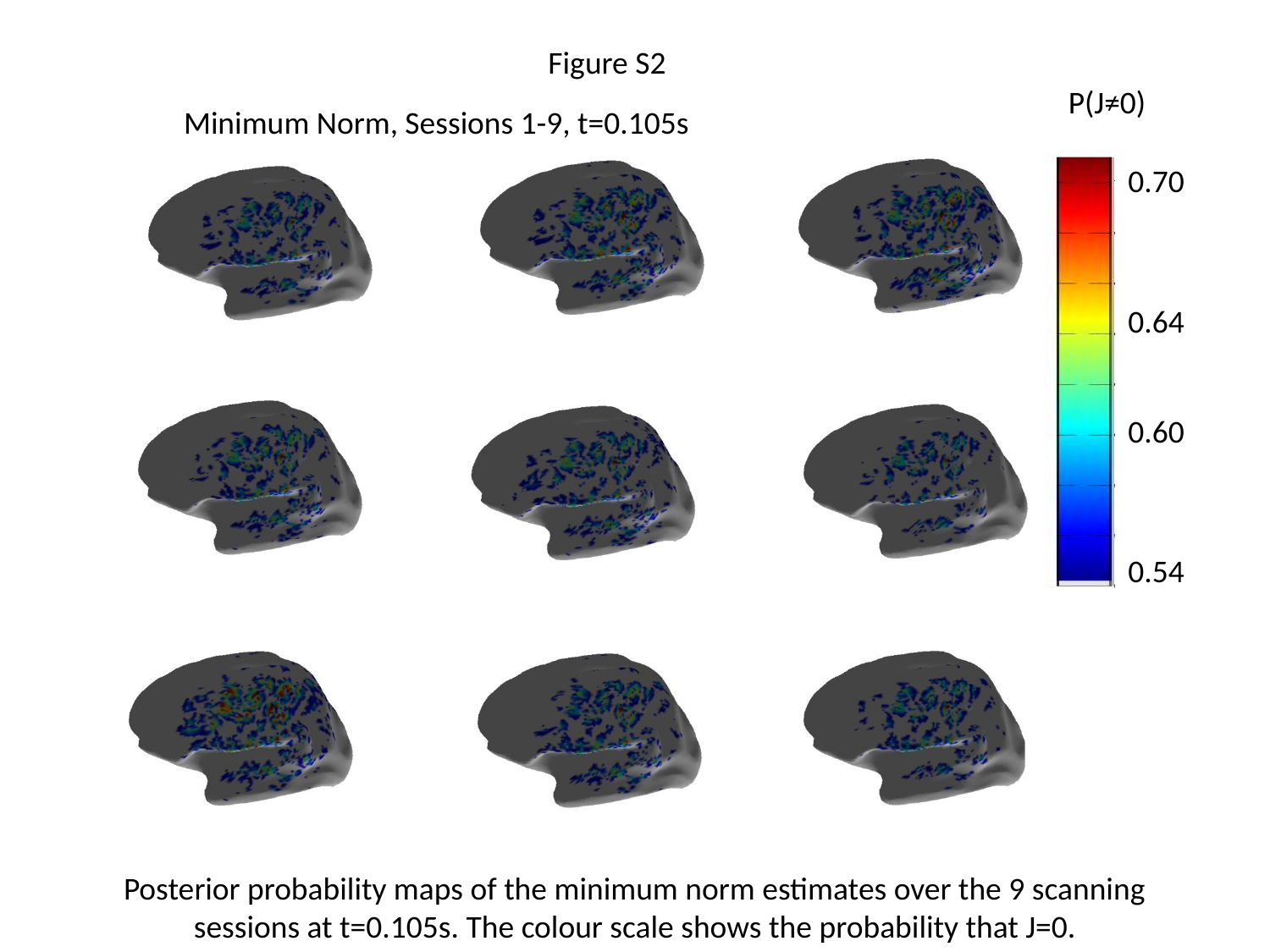

Figure S2
P(J≠0)
Minimum Norm, Sessions 1-9, t=0.105s
0.70
0.64
0.60
0.54
Posterior probability maps of the minimum norm estimates over the 9 scanning sessions at t=0.105s. The colour scale shows the probability that J=0.
